# Genotype-Mitral Valve Phenotype Correlations in Marfan Syndrome With *FBN1* Pathogenic Variants

**DOI:** 10.1016/j.jacadv.2022.100149

**Published:** 2022-12-14

**Authors:** Clémence Delhomme, Olivier Milleron, Florence Arnoult, Gabriel Delorme, Ludivine Eliahou, Sabrine Jadoui, Pauline Arnaud, Nadine Hanna, Catherine Boileau, Guillaume Jondeau

Genotype-phenotype correlations in the Marfan syndrome (MFS) population with *FBN1* pathogenic variants have been reported^1^. Premature termination codon (PTC) variants, responsible for haploinsufficiency and, therefore, a decreased amount of fibrillin-1 production within the cells, were associated with a higher frequency of aortic events. Moreover, among patients with in-frame variants (IFVs) responsible for an alteration in the amino acid content of fibrillin-1, aortic events and survival depended on the effect of the pathogenic variant on the cysteine content of fibrillin-1. Variants responsible for a loss of cysteine in the fibrillin-1 molecule were associated with more aortic events, whereas variants responsible for an increase in the cysteine content were associated with fewer aortic events, while variants with no effect on the cysteine content were associated with intermediate risk.

Mitral valve prolapse (MVP) has a great prevalence in the MFS population, as high as 40% in some series^2^. Recently, Demolder et al^3^ reported an association among mitral annular disjunction (MAD), arrhythmic events, and mitral valve surgery in 142 MFS patients, underlining the importance of MVP in this population. Arrhythmic events have been recognized as possible causes of sudden death in the MFS population^4^. However, no genotype-phenotype correlation regarding mitral valve features has been reported in the literature.

From 2015 to 2017, we included 250 MFS patients with a *FBN1* pathogenic gene variant, older than 14 years, without a thoracic surgery, and seen in the French reference center. Mitral valve was studied precisely by using a standardized transthoracic echocardiographic protocol. MVP was defined by a mitral leaflet displacement of ≥2 mm in systole into the left atrium, beyond the mitral annular plane, according to Levine definition. MAD was measured in the parasternal long-axis view at end-systole, as the distance between the mitral annulus and the basal portion of the left ventricular inferolateral wall. We sought the associations between genotype and mitral valve features, according to the presence of PTC variants or IFVs. IFVs were divided according to the impact on the cysteine content of fibrillin-1: 99 patients (39.6%) had PTC variants, and 151 patients (60.4%) had IFVs. Among patients with an IFV, 27 had missense variants that substitute for a cysteine (+Cys), 49 had missense variants that substitute a cysteine for another amino acid (−Cys), and 75 had IFVs not modifying the cysteine content of fibrillin-1 (noCys).

**Figure 1:**
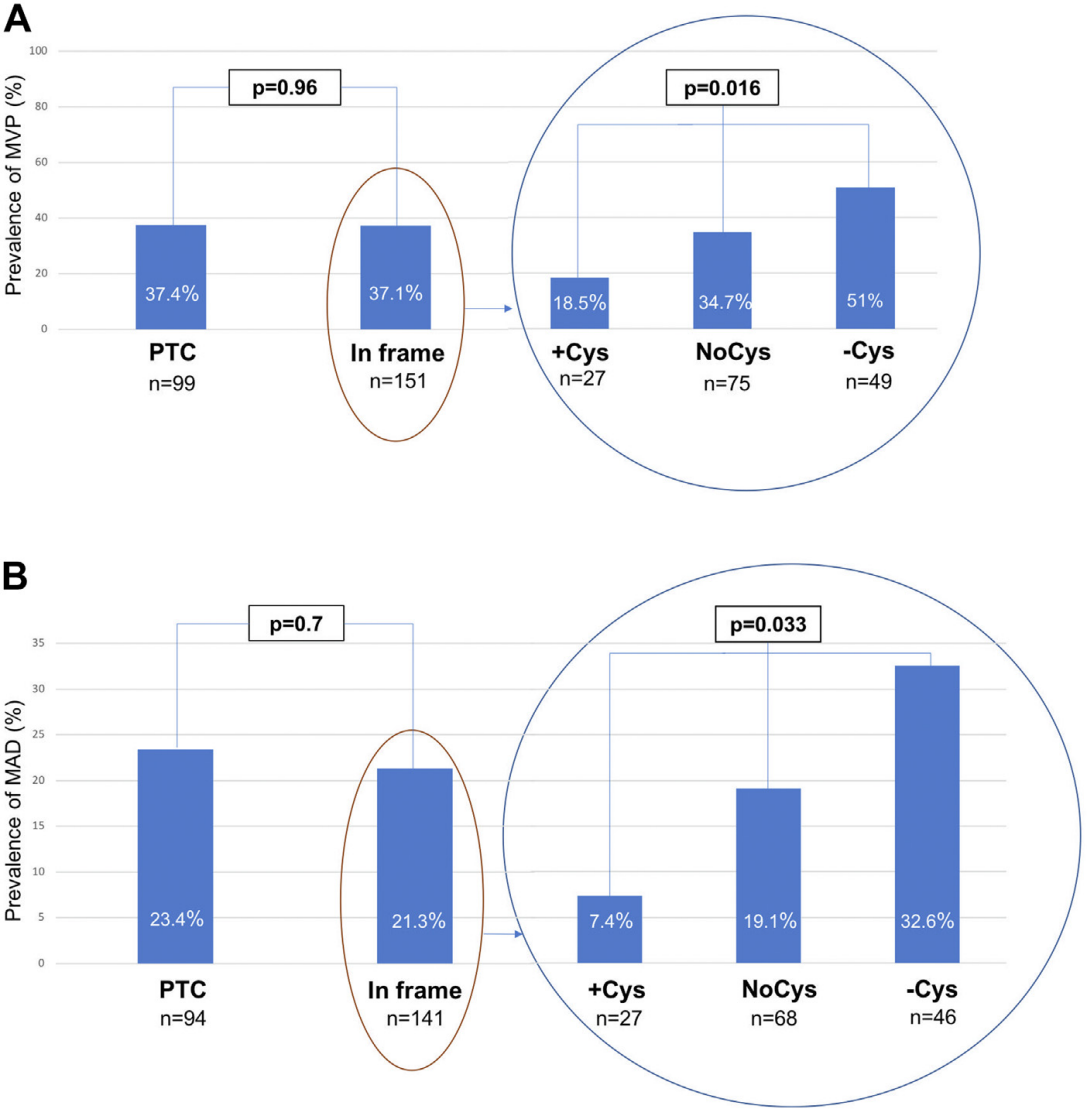
Genotype-Mitral Valve Phenotype Correlations in Marfan Syndrome Prevalence of mitral valve prolapse (MVP) **(A)** and mitral annular disjunction (MAD) **(B)** according to the presence of premature termination codon (PTC) variants or in-frame variants/according to the impact on the cysteine content of fibrillin-1 among patients with in-frame variants.

Overall, the prevalence of MVP (93/250, 37.2%) was similar in patients with PTC and IFV (37/99, 37.4% vs 56/151, 37.1%; *P* = 0.96). In addition, prevalence of MAD (52/235; 22.1%) was similar in patients with PTC and IFV (22/94, 23.4% vs 30/141, 21.3%; *P* = 0.70). However, among patients with IFV, patients with the −Cys variants had significantly more MVP than patients with noCys variants and patients with +Cys variants (25/49, 51%, vs 26/75, 34.7%, vs 5/27, 18.5%, respectively; *P* = 0.016). Prevalence of MAD was also more important in patients with −Cys variants than patients with noCys variants and patients with +Cys variants (15/46, 32.6%, vs 13/68, 19.1%, vs 2/27, 7.4%, respectively; *P* = 0.033) (Figure 1).

Our cohort study is the first study reporting a genotype-phenotype correlation for MVP and MAD, in the MFS population. The higher prevalence of MVP and MAD was observed in the −Cys variants subgroup, a group also associated with more aortic events^1^. However, PTC variants were not associated with a higher prevalence of MAD and MVP, while the incidence of aortic events is maximal in this subgroup. Lastly, we observed a lower prevalence of MVP and MAD in the +Cys variants subgroup, a group also associated with a lower prevalence of aortic events. These observations may suggest different pathophysiologic mechanisms, with different arrhythmic and aortic risks according to defined genetic subgroups, which could allow for individualized risk evaluation and therapy. However, these suggestions need to be confirmed in a prospective study with a larger population.
